# Silicone-Based Thermally Conductive Gel Fabrication via Hybridization of Low-Melting-Point Alloy–Hexagonal Boron Nitride–Graphene Oxide

**DOI:** 10.3390/nano13030490

**Published:** 2023-01-25

**Authors:** Peijia Chen, Xin Ge, Zhicong Zhang, Shuang Yin, Weijie Liang, Jianfang Ge

**Affiliations:** 1 College of Chemistry and Chemical Engineering, Zhongkai University of Agriculture and Engineering, Guangzhou 510225, China; 2 School of Materials and Energy, Guangdong University of Technology, Guangzhou 510006, China; 3 College of Resources and Environment, Zhongkai University of Agriculture and Engineering, Guangzhou 510225, China; 4 School of Materials Science and Engineering, Northwestern Polytechnical University, Xi’an 710072, China

**Keywords:** thermally conductive gel, low-melting-point alloy, thermal contact resistance, thermal interface material

## Abstract

Thermal contact resistance between the microprocessor chip and the heat sink has long been a focus of thermal management research in electronics. Thermally conductive gel, as a thermal interface material for efficient heat transfer between high-power components and heat sinks, can effectively reduce heat accumulation in electronic components. To reduce the interface thermal resistance of thermally conductive gel, hexagonal boron nitride and graphene oxide were hybridized with a low-melting-point alloy in the presence of a surface modifier, humic acid, to obtain a hybrid filler. The results showed that at the nanoscale, the low-melting-point alloy was homogeneously composited and encapsulated in hexagonal boron nitride and graphene oxide, which reduced its melting range. When the temperature reached the melting point of the low-melting-point alloy, the hybrid powder exhibited surface wettability. The thermal conductivity of the thermally conductive gel prepared with the hybrid filler increased to 2.18 W/(m·K), while the corresponding thermal contact resistance could be as low as 0.024 °C/W. Furthermore, the thermal interface material maintained its excellent electric insulation performance, which is necessary for electronic device applications.

## 1. Introduction

Thermal management is crucial in electronic cooling due to constant increases in the power density of electronic devices. Impeded heat transfer capacity due to a bump or corrugated interface between electronic components and radiators severely reduces the reliability of electronic devices. Eliminating the air gap between the interfaces and providing more heat conduction points will reduce thermal contact resistance (TCR) and improve heat transfer efficiency [1,2]. Thermal interface materials (TIM), with properties such as thermal conductivity, interface wettability, durability, etc., are used to fill the interface gap and are required to have excellent heat dissipation effects, thereby improving reliability [3]. Thermally conductive gel (TCG) is a new type of TIM with these characteristics, particularly in terms of durability for weak oil pump-out, compared with thermally conductive grease. Conventionally, TCG is prepared using reactive crosslinking silicone oil and heat-conductive particles [4]. Some key issues must be addressed to obtain excellent thermal conductivity for TIM.

First, the thermally conductive properties of TCG mainly depend on the filling materials. When fillers are incorporated into the polymer matrix, they form a thermally conductive network, thus improving the composite’s thermal conductivity. Graphene has a significantly higher inherent thermal conductivity than carbon nanotubes, making it a suitable thermally conductive filler [5,6]. Adding graphene nanosheets (10 vol%) to pure gel sheets increased the thermal conductivity by 90% [7]. However, graphene’s electrical conductivity limits its applications, especially in electronic packaging where electronic insulation is required. Graphene oxide (GO) prepared following the Hummer method has electronic insulation properties due to severe functionalization of the conjugate network [8]. Hexagonal boron nitride (h-BN), which has a similar crystal structure to graphene, is attractive as an alternative filler due to its excellent thermal conductivity, electrical insulation, and high thermal stability. Compared to an epoxy resin substrate, its thermal conductivity coefficient can be significantly improved through hybridization with h-BN and GO fillers [9]. That is, the hybridization of different fillers is one of the possible ways to improve the performance of fillers and TIM.

Second, filler surface modification is performed to improve filler dispersion in the matrix and achieve better hybrid filler interface connection to improve the composite’s thermal conductivity. As previously reported, the hybrid structure of surface-modified BN and GO was synthesized by surface modification of the filler, which has a stable interface [10]. The silver nanoparticle served as a bridging agent for the phonon transport of the hybrid structure. BN–Ag–GO is an effective thermally conductive filler in epoxy matrices [11]. Referring to this scheme, phase change materials with tunable melting temperatures provide a new design for thermal energy storage applications. Low-melting-point alloy (LMPA) has recently emerged as one of the directions of thermal conductivity reinforcement [12,13]. It has medium-high thermal conductivity and many applications, particularly excellent thermal physical properties, including wettability and adhesion, which are essential for surface conformability with little or no thermal resistance [14]. LMPA can be used as a new electronic packaging material by introducing it into a polyvinylidene fluoride matrix [15]. To date, LMPA is used for thermal management in various applications, such as desktop computers, light-emitting diodes, electronic cooling, and biological heat transfer [16,17]. By combining LMPA and BN, Ge Xin et al. found that the thermal conductivity of thermal grease reached 1.8 W/(m·K) and effectively reduced its TCR from 13.8 to 0.547 °C/W [18]. Due to the poor surface energy of BN, its microspheres need to be modified by dopamine to form a compact inorganic network structure with LMPA. Adding LMPA can improve the coherence of the inorganic filler in the polymer matrix, moderate agglomeration in the polymer matrix, and improve the thermal conductivity of the polymer material.

Electronic equipment must have efficient heat dissipation capacity and maintain a certain insulation performance in various scenarios to ensure long-term safe operation [19]. The published literature suggests that LMPA-based composites may have higher electrical conductivity than those based on BN or carbon nanotubes [12,20]. To improve the thermal conductivity of TCG and ensure electrical insulating properties, we mixed LMPA with an inorganic electrically insulating material (BN) to prepare thermally conductive fillers. During filler modification [15,21], LMPA melts after the temperature reaches the melting point. As a bridging agent, LMPA, combined with other nonmetallic fillers, forms a micro continuous thermally conductive network, effectively reducing the TCR of TCG [22]. If LMPA is applied to interfaces, inappropriate surface wettability may lead to unacceptable performance deterioration [14]. In this article, we focus on the recent results of the research group.

## 2. Experiment Section

### 2.1. Materials

A eugenol-synthesized crosslinker was constructed, according to the literature [23]. Double-end vinyl silicone oil (VS1000, 1000 mPa·s, 0.12 mmol/g) was provided by Ambia Specialty Silicone Co., Ltd. (Waukegan, IL, USA). Liquid Pt catalyst (5000 ppm) and ethanol (AR) were supplied by Tianjin Comio Chemical Co., Ltd. (Tianjin, China). Humic acid (HA) was purchased from Shanghai Maclin Biochemical Technology Co., Ltd. (Shanghai, China). LMPA was purchased from Dongguan Wochang Metal Products Co., Ltd. (Dongguan, China). h-BN (200 nm) and GO were obtained from Zhejiang Yamei Nanotechnology Co., Ltd. (Jiaxing, China). and Zibo Jingyi Ceramic Technology Co., Ltd. (Zibo, China), respectively.

### 2.2. Sample Preparation

**Figure 1:**
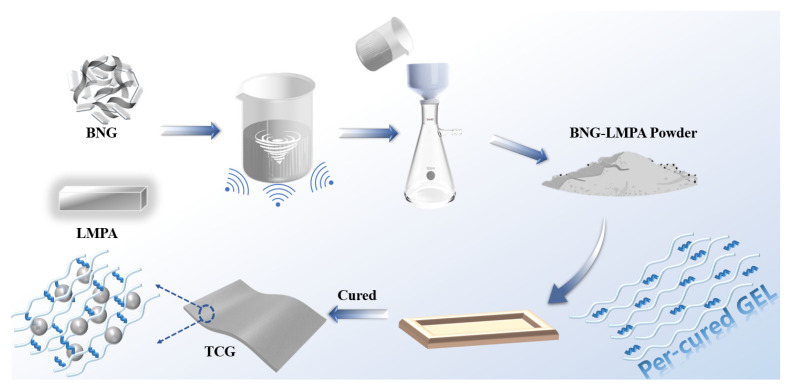
Schematic diagram of the preparation process of BNG-LMPA/SG.

First, h-BN and GO were modified by HA to reduce their surface energy and improve their interaction with LMPA. Modification will be introduced in the future. Furthermore, 95 wt.% h-BN and 5 wt.% GO were dispersed in ethanol under ultrasonic stirring for 4 h and kept at room temperature for 24 h without stirring. After filtration, h-BN@GO (BNG) was obtained by repeatedly washing with deionized (DI) water and vacuum drying at 80 °C for 24 h. Then, using DI water as the medium, LMPA was liquefied at a constant temperature of 80 °C. In consideration of interface wettability and adhesion of LMPA, BNG with mass ratios of 4:1, 2:1, 1:1, and 1:2 was redispersed in water. Ultrasonic stirring was performed again for 3 h to create the BNG and LMPA composite. Finally, the composite powder was filtered and dried at 50 °C. The subscript represents the ratio of BNG to LMPA; for example, BNG_4_LMPA_1_ indicates that the mass ratio of BNG to LMPA is 4:1. The operation was conducted as depicted in Figure 1.

The resulting BNG–LMPA was then added to silica gel (SG) prepared in advance, and the low modulus and high elasticity of SG were used to prepare high-performance TCG. In contrast, the same ratio of BN–LMPA-based TCG was also synthesized.

### 2.3. Characterization

An X-ray diffraction analyzer (XRD, Bruker, Germany) was used to analyze the composition of the material and the structure of atoms or molecules within the material. Scanning electron microscopy (FEI Co., Hillsboro, USA) was performed to examine the morphology of the thermally conductive fillers. During heating, micromorphological changes in BNG–LMPA were observed using an MP41 optical microscope (Mingmei Photoelectric Technology Co., Guangzhou, China). The LMPA’s effect on the composite powder’s latent heat was investigated using a Q2000 differential scanning calorimetry (DSC) instrument (TA Instruments Inc., Newcastle, USA). TCG’s thermal conductivity was measured using a transient short hot-wire technique with a TC-3000 TC meter (Xiaxi Electronic Technology Co., Xi’an, China), and thermal resistance was measured using an LW-9389 thermal resistance tester (Ruineng Technology Co., Taiwan, China). Heat diffusion ability was evaluated using a TiS10 Fluke infrared thermal imager. The breakdown voltage and volume resistance of TCG were tested using a ZJC-50KV voltage breakdown tester (Beijing AvIC Times Instrument Equipment Co., Beijing, China) and BEST-303 volume resistivity tester (Beijing Beiguang Jing Instrument Equipment Co., Beijing, China), respectively.

## 3. Results and Discussion

### 3.1. Microstructure and Element Distribution of the Hybrid Filler

**Figure 2:**
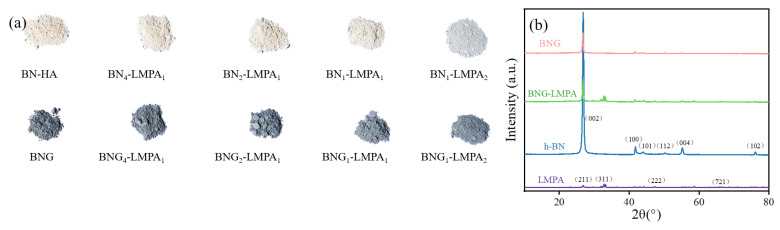

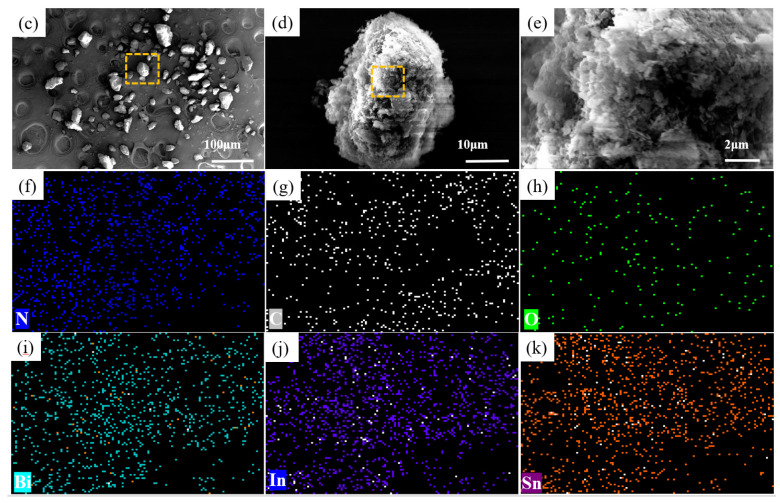
(**a**) Photo of BN-LMPA and BNG-LMPA; (**b**) XRD spectra of h-BN, BNG, LMPA, and BNG-LMPA; (**c**–**e**) SEM morphology of BNG_1_LMPA_2_ and its elemental analysis: (**f**–**k**) N, C, O, Bi, In, and Sn.

As depicted in Figure 2a, BN–LMPA and BNG–LMPA appeared white and gray, respectively. h-BN, BNG, LMPA, and BNG–LMPA were characterized by XRD. The diffraction peaks (002), (100), (101), (102), (004), and (112) shown in Figure 2b were the XRD characteristic diffraction peaks of h-BN and BNG. The XRD characteristic peaks of LMPA were Sn (29.8°, 32.1°), Bi (36.3°, 47.4°), and In (58.7°, 66.4°) [24]. The presence of Ga in LMPA was not directly detected by XRD, thus indicating that the Ga signal was too weak or Ga was absent in LMPA. Due to the Ga-driven degradation of many metal microelectronic components, the corrosiveness and toxicity of Ga-based LMPA affect its use. An LMPA without Ga, an inorganic insulating filler, and polymer matrix three-component composites are more likely to provide a cheaper, noncorrosive solution [25]. The XRD spectra of BN–LMPA and BNG–LMPA showed that the strength of (100), (101), (004), and (102) crystal planes gradually decreased, and weak characteristic diffraction peaks of LMPA appeared, indicating that h-BN and BNG combined well with LMPA.

HA contains several active functional groups, such as carboxyl and phenolic hydroxyl groups, which are excellent ceramic dispersants. Its hydrophobic properties reduced the surface free energy of the powder. Thus, the BN and GO particles modified by the HA surface repelled each other, and the dispersed particles were hindered by space, ensuring that the stability of the dispersion system was maintained. After modification, GO combined with BN by winding and surface action to form an elliptical sphere (BNG) (Figure S1). As shown in Figure 2c–e, BNG was compounded with LMPA through an independently designed preparation method to maintain the shape of the elliptical particulate matter of BNG. It has been proven that LMPA does not alter the structure of BNG but can form a unique structure, with BNG as the skeleton, by adhering to or even penetrating BNG. Element distribution analysis of BNG_1_LMPA_2_ revealed that Figure 2f–k elements (N, C, O, Bi, In, and Sn) were uniformly distributed in the whole space, indicating that LMPA and BNG were uniformly recombined in the microstructure.

### 3.2. Phase Change of LMPA in Hybrids

**Figure 3:**
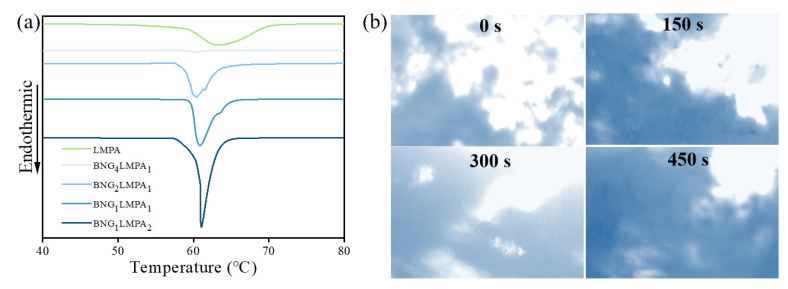
(**a**) DSC curves of BNG-LMPA; (**b**) Morphological changes of BNG_1_LMPA_2_ on the hot plate.

The latent heat of phase change and phase change temperature are two of the most vital thermal physical energy parameters of phase change materials that influence their applicability. TIM is made from a phase change material, which can rapidly absorb heat and cool down electronic devices when heated. The DSC test on BNG–LMPA was used to investigate the influence of the different fillers and their ratios on the melting point. Figure 3a shows that the phase transition temperature range of LMPA was 47–72 °C, which was within the heating temperature range of general electronic devices, and the maximum absorption peak was reached at 67 °C. When BNG was combined with LMPA, the maximum absorption peak temperature was reduced, and the LMPA melting range decreased [18]. The energy absorbed by melting was proportional to the LMPA content in the composites, and the latent heat of phase change of the two composites increased with increasing LMPA content. Since BNG has a higher heat transfer capacity than LMPA [K_Sn_ = 67.0 W/(m·K), K_Bi_ = 7.9 W/(m·K), and K_In_ = 82.0 W/(m·K)] when it is uniformly mixed with LMPA at the microscopic level, heat can be transferred quickly to reach the initial melting temperature of LMPA. The results showed that BNG–LMPA/SG could rapidly absorb heat, thereby protecting electronic devices before the temperature reached the limit.

The spreading (interfacial bonding) process of BNG_1_LMPA_2_ under a certain pressure and heating program (5 °C/s) was observed using the transmission light of an optical microscope. As shown in Figure 3b, BNG_1_LMPA_2_ blocked the light, so the sample part appeared in shadow while other parts without the sample appeared white. During heating, the TCG temperature reached the melting point of LMPA at approximately 150 s, and the image changed to varying degrees. BNG_1_LMPA_2_ also underwent a phase transformation at 150 s. Under external pressure, LMPA spread easily and had a large diffusion range. At 450 s (80 °C), the black area gradually expanded, at which point LMPA infiltrated from the inside of BNG and covered the entire slide surface. This indicated that BNG_1_LMPA_2_ had good wettability in TIM, enabling it to rapidly transmit heat flow and effectively protect heating devices.

### 3.3. Thermal Conductivity of BN–LMPA/SG and BNG–LMPA/SG

**Figure 4:**
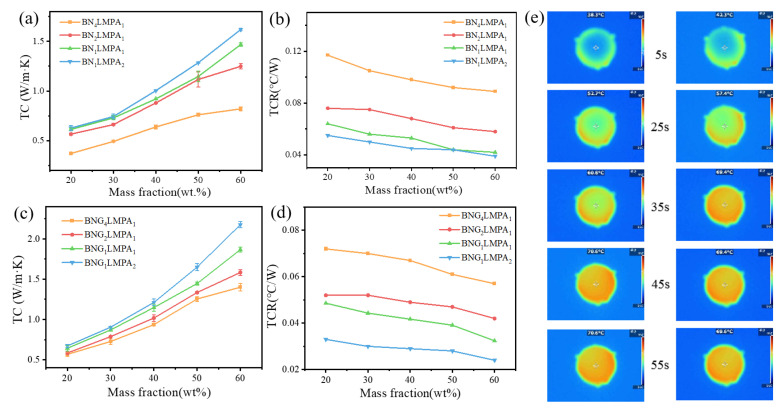
TC and TCR of (**a**,**b**) BN-LMPA and (**c**,**d**) BNG-LMPA; (**e**) Infrared thermography of BN_1_LMPA_2_/SG (left) and BNG_1_LMPA_2_/SG (right).

The thermal conductivity of composites is mainly determined by the heat transfer capacity of the filler, thermal network density, and TCR. BN–LMPA and BNG–LMPA of different proportions were prepared to compare the effect of the LMPA content on the thermal conductivity of TCG with or without GO. On the basis of h-BN and BNG, LMPA was added to prepare different proportions of BN-LMPA and BNG-LMPA. From Table 1, it can be seen that h-BN with GO initially improved the thermal conductivity of TCG, while LMPA further improved the thermal conductivity of TCG. Figure 4a,b indicates the thermal conductivity and TCR of BN–LMPA/SG, respectively. When the LMPA content was low, the thermal conductivity increased slowly with increasing BN_4_LMPA_1_ filling amount. In this case, BN_4_LMPA_1_, and the surrounding matrix had high TCR and interfacial phonon scattering, which was not conducive to heat transfer. When the LMPA content was further increased, the contribution of the composite filler to thermal conductivity was significantly improved. When the filling amount of BN_1_LMPA_1_ was 60 wt.%, the thermal conductivity reached 1.47 W/(m·K), which was approximately 8.7 times that of the pure gel matrix (0.17 W/m·K). An appropriate amount of LMPA promoted the formation of a heat conduction path; thus, it synergistically enhanced the thermal conductivity of TCG with h-BN. When the filling amount of BN_1_LMPA_2_/SG was 60 wt.%, the thermal conductivity reached 1.62 W/(m·K) and the TCR decreased to 0.039 °C/W, which was 97% higher and 128% lower than that of BN_4_LMPA_1_/SG, respectively. This indicated that BN_1_LMPA_2_-filled composites had a certain TC enhancement, particularly TCR reduction.

Figure 4c,d indicates the thermal conductivity and TCR curves of BNG–LMPA/SG, respectively. When the filling amount of BNG_2_LMPA_1_/SG was 60 wt.%, the thermal conductivity was 1.59 W/(m·K) and the TCR decreased to 0.042 °C/W, which was similar to that of BN_1_LMPA_2_. The preliminary results showed that the presence of GO resulted in a more significant contribution of BNG–LMPA to the composite’s thermal conductivity than BN–LMPA. When used as TIM, LMPA exchanged heat flow between the thermal filler and matrix, significantly improving the composite’s thermal conductivity. Meanwhile, BNG–LMPA/SG hot pressing formed a coherent network structure, which reduced the interface thermal resistance. The maximum thermal conductivity of BNG_1_LMPA_2_/SG filled with 60 wt.% was approximately 2.18 W/(m·K) and the TCR was as low as 0.024 °C/W, which increased by 35% and decreased by 63%, respectively, when compared with BN_1_LMPA_2_/SG filled with the same amount.

To visualize the heat transfer effects of BN_1_LMPA_2_/SG and BNG_1_LMPA_2_/SG, the two composite materials with 60 wt.% filling volume were uniformly coated on the slide, and the temperature response difference was recorded by infrared thermography. Figure 4e depicts the heat transfer efficiency of BN_1_LMPA_2_/SG and BNG_1_LMPA_2_/SG. At 5 s, the surface temperature of BN_1_LMPA_2_/SG (left) was 38.3 °C, and it reached equilibrium after approximately 45 s, similar to BNG/SG, but at a lower equilibrium temperature. Alternatively, the surface temperature of BNG_1_LMPA_2_/SG rose rapidly to 42.3 °C in 5 s, and it only took 35 s to reach equilibrium, with a lower equilibrium temperature than that of BN_1_LMPA_2_/SG. This was attributed to the fact that BNG_1_LMPA_2_ could accelerate heat emission and had a higher heat dissipation effect. Lattice vibration (phonon) is the primary mechanism of heat conduction of nonmetallic materials, but it contributes less to the heat conduction of metallic materials. Here, LMPA can provide a pathway for electron heat conduction. As the temperature rises, lattice vibration and free-electron movement combine to form a new equilibrium state, increasing heat transfer via phonons and electrons [26]. Based on the above analysis, BNG_1_LMPA_2_/SG can play a better heat transfer role in the system due to the actions of LMPA.

**Figure 5:**
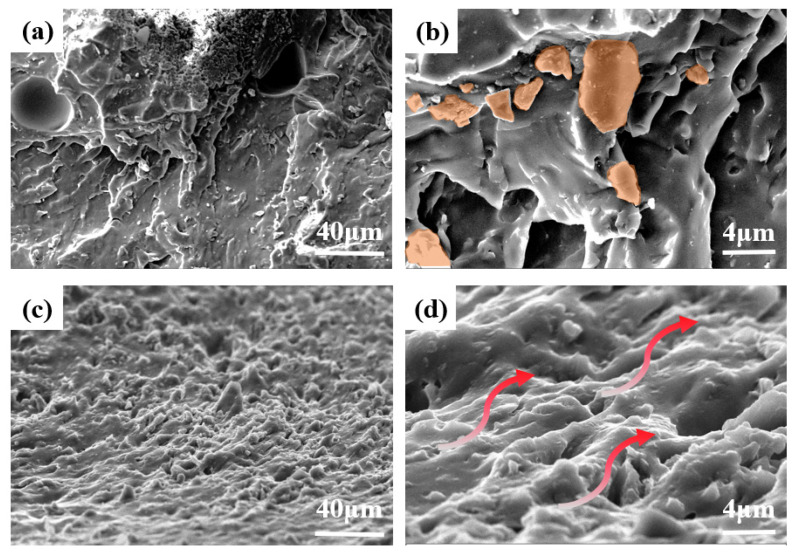
Cross-section morphology of (**a**,**b**) BN_1_LMPA_2_/SG and (**c**,**d**) BNG_1_LMPA_2_/SG.

The formation of an effective three-dimensional seepage network of heat flow via the synergistic effect is the key criterion for determining the thermal conductivity of composite materials [27]. As shown in Figure 5a, BN_1_LMPA_2_ was less evenly distributed in TCG, where the blend filler of LMPA and h-BN had made a relatively limited contribution to the thermal conductivity of TCG. When used as TIM, the heat flow could not be effectively transmitted between the thermally conductive filler due to heat flow network defects, resulting in poor thermal conductivity. Meanwhile, as shown in Figure 5b, LMPA adhered to the surface of h-BN and GO in the presence of HA, and the hybridization interaction between LMPA and h-BN was significantly improved. Figure 5c,d demonstrates the microscopic morphology of BNG_1_LMPA_2_/SG. The continuity of BNG/SG and BN_1_LMPA_2_/SG was stronger, and the thermally conductive network was more complete. This indicated that the melting flow of LMPA inside BNG_1_LMPA_2_/SG, which ran through the BNG interior, connected adjacent fillers in the matrix to form a new heat conduction path, which could still be maintained after the temperature was cooled. The comparison between h-BN and BNG–LMPA/SG showed that the discontinuity defect of h-BN in TCG was compensated by LMPA, with BNG–LMPA/SG contributing more significantly to reducing the interface thermal resistance.

### 3.4. Electric Insulation Performance of TCG

**Figure 6:**
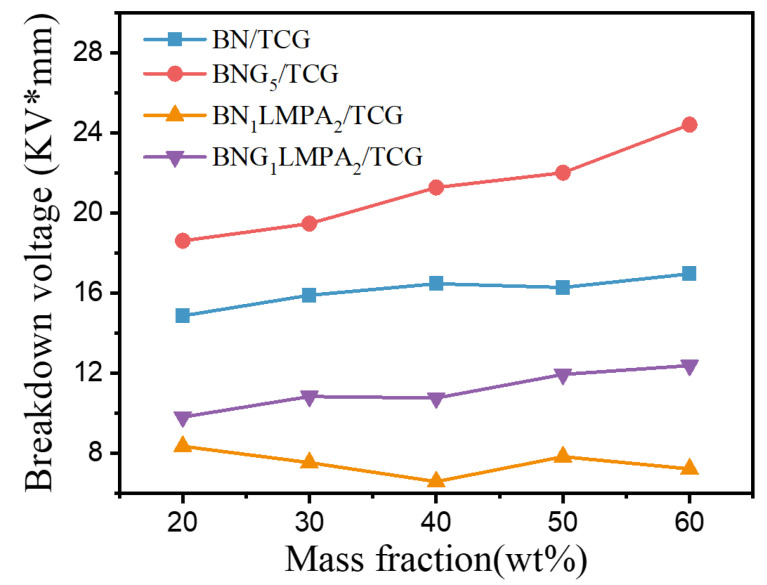
Breakdown voltages of different TCG.

In addition to heat accumulation, a bad electromagnetic wave caused by electronic equipment is also a potential hazard [28]. Therefore, TIM with mutually multifunctional properties, such as a high dielectric constant, high breakdown strength, and high TC, are preferred. The structure of h-BN is similar to that of electrically conductive graphene or graphite, except no free electron exists. GO prepared following the Hummers method is functionalized due to the conjugate network, showing insulation characteristics [29,30]. The conductivity of materials is usually expressed by resistivity or conductivity independent of size. In this experiment, two methods were selected to test the conductivity of TCG, namely, breakdown voltage (Figure 6) and volume resistivity (Table 2).

GO and h-BN take advantage of the excellent inplane thermal conductivity of two-dimensional fillers to reduce electrical conduction along the thickness direction, which effectively translates into high thermal conductivity and breakdown strength of composites. Compared with BN/SG, the volume resistivity and breakdown voltage of BNG/SG were enhanced. The addition of LMPA inhibited the insulation performance of BN_1_LMPA_2_/SG, in which the volume resistivity decreased by an order of magnitude and the breakdown voltage decreased by approximately 50%. The BNG_1_LMPA_2_ composite filler prepared by combining BNG with LMPA still had high volume resistivity and the breakdown voltage was higher than that of BN_1_LMPA_2_/SG, which met the requirements of insulating occasions. Compared with the pristine polymer, BNG_1_LMPA_2_/SG exhibited multifunctional properties under various synergistic mechanisms, including high thermal conductivity, low thermal resistance, and medium insulation performance.

## 4. Conclusions

TIM increased the heat transfer path and improved the heat dissipation efficiency by serving as a “bridge” between the heating device and the heat sink. LMPA was introduced in the BNG-based TCG for improved thermal management. LMPA and BNG were uniformly compounded on a nanometer scale based on a self-designed preparation. Using the phase change characteristics of BNG-loaded LMPA, an effective heat transfer path was fabricated, resulting in significantly improved thermal conductivity and TCR of the TIM. More specifically, the thermal conductivity of BNG_1_LMPA_2_/SG increased to 2.18 W/(m·K), which was 12.8 times that of the matrix, while the TCR decreased to 0.024 °C/W, which was 63% lower than that of BN_1_LMPA_2_/SG with the same filling amount. Hybridization of a composite of LMPA–BN–OG in the presence of HA can provide a strategy for ensuring TCG with high thermal conductivity and low thermal resistance while maintaining volume resistivity and breakdown voltage within a specific range. BNG-LMPA, a ternary hybrid material, provided a significant contribution to thermal conductivity, which is conducive to subsequent research of multi-hybrid materials. Additionally, this work provides guidance for the comprehensive improvement of TIM thermal management performance from the aspects of building an efficient thermal conduction path and improving the interface wetting effect.

## Figures and Tables

**Table 1:** Thermal conductivity of different TCG.

Dosage/wt.%	Thermal Conductivity/(m·K)			
	BN/SG	BNG/SG	BN_1_LMPA_2_/SG	BNG_1_LMPA_2_/SG
20	0.29	0.53	0.63	0.67
30	0.34	0.72	0.75	0.91
40	0.47	0.91	1.01	1.22
50	0.56	1.21	1.30	1.65
60	0.61	1.39	1.62	2.17

**Table 2:** Volume resistivity of different TCG.

Dosage/wt.%	Volume Resistance (Ω·cm)			
	BN/SG	BNG/SG	BN_1_LMPA_2_/SG	BNG_1_LMPA_2_/SG
20	1.09 × 10^15^	8.19 × 10^15^	5.11 × 10^14^	1.38 × 10^15^
30	1.08 × 10^15^	1.73 × 10^16^	5.31 × 10^14^	1.86 × 10^15^
40	7.74 × 10^15^	3.57 × 10^16^	6.22 × 10^14^	2.39 × 10^15^
50	1.48 × 10^16^	6.91 × 10^16^	8.53 × 10^14^	2.67 × 10^15^
60	2.01 × 10^16^	9.48 × 10^16^	1.21 × 10^15^	3.72 × 10^15^

## Data Availability

The data presented in this study are available on request from the corresponding author.

## Supplementary Materials

The following supporting information can be downloaded at: https://www.mdpi.com/article/10.3390/nano13030490/s1, Figure S1: (a) SEM image, (b) XPS spectra and (c) FT-IR spectra of BNG.
