# Supplementary material for: Silicone-Based Thermally Conductive Gel Fabrication via Hybridization of Low-Melting-Point Alloy–Hexagonal Boron Nitride–Graphene Oxide
Source: Nanomaterials (Basel). 2023 Jan 25;13(3):490. doi: 10.3390/nano13030490 (PMC9920594; doi:10.3390/nano13030490)
Supplement: Supplementary file 1 [file nanomaterials-13-00490-s001.zip › nanomaterials-2165929-supplementary.pdf]

nanomaterials

MDPI

*Article*

# Silicone-Based Thermally Conductive Gel Fabrication via Hybridization of Low-Melting-Point Alloy–Hexagonal Boron Nitride–Graphene Oxide

**Peijia Chen [1,†], Xin Ge [2,†], Zhicong Zhang [1], Shuang Yin [3], Weijie Liang [4] and Jianfang Ge [1,*]**

1 College of Chemistry and Chemical Engineering, Zhongkai University of Agriculture and Engineering, Guangzhou 510225, China
2 School of Materials and Energy, Guangdong University of Technology, Guangzhou 510006, China
3 College of Resources and Environment, Zhongkai University of Agriculture and Engineering, Guangzhou 510225, China
4 School of Materials Science and Engineering, Northwestern Polytechnical University, Xi'an 710072, China
* Correspondence: gejianfang@zhku.edu.cn
† These authors contributed equally to this work.

In Figure S1b, the spectrum of BN shows two primary peaks related with the typical vibrations of the BN framework: in-plane BN stretching at 1391 $cm^{-1}$ and out-of-plane BN bending mode at 806 $cm^{-1}$. There are three main peaks centered at 1380, 1680 and 3470 $cm^{-1}$ in GO. The peak at 1680 $cm^{-1}$ corresponds to the vibrational mode of the ketone (-C=O) groups. The peak observed at 1380 $cm^{-1}$ is assigned to a C-O vibrational mode. The peak at 3470 $cm^{-1}$ denotes C-OH stretching. All characteristic peaks exist in BNG and no new peak generate, means BN combined with GO physically.

In Figure S1c, C 1s peak (285.08 eV) and O 1s peak (533.08eV) of GO, and B 1s (397.08eV) and N 1s (190.08eV) of BN are all maintained in BNG, indicating that their chemical structure had not change.

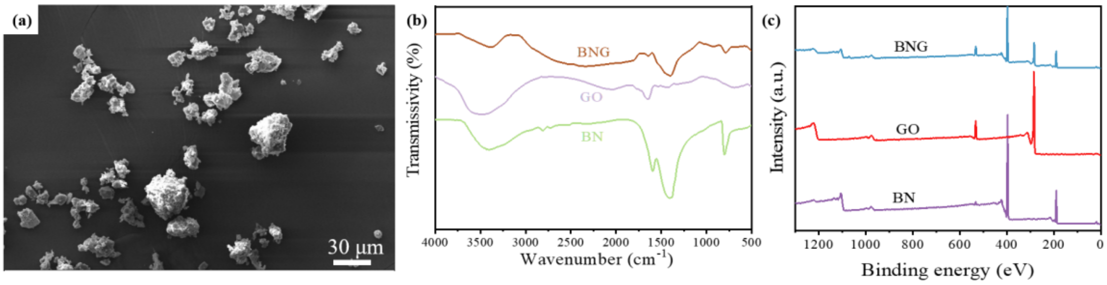


**Figure S1.** (**a**) SEM image, (**b**) XPS spectra and (**c**) FT-IR spectra of BNG.
